# Recurrent Outbreak of *Campylobacter jejuni* Infections Associated with a Raw Milk Dairy — Pennsylvania, April–May 2013

**Published:** 2013-08-30

**Authors:** André Weltman, Allison H. Longenberger, Mària Moll, Lydia Johnson, Judy Martin, Amanda Beaudoin

**Affiliations:** Pennsylvania Dept of Health; Pennsylvania Dept of Agriculture; EIS Officer, CDC

During May 2013, the Pennsylvania Department of Health investigated an outbreak of campylobacteriosis among consumers of raw (unpasteurized) milk from a dairy certified by the Pennsylvania Department of Agriculture (PDA) to sell raw milk onsite, at retail stores, and at off-farm pick-up sites. Investigation by the Pennsylvania Department of Health and PDA identified six confirmed and two probable cases of campylobacteriosis associated with raw milk from the dairy. A confirmed case was defined as laboratory-confirmed campylobacteriosis in a person who drank the dairy’s raw milk. A probable case was defined as diarrheal illness without laboratory confirmation in a person who had consumed the dairy’s raw milk and was linked to a confirmed case. Four cases involved children aged ≤18 years. PDA identified *Campylobacter* in bulk tank and retail milk samples from the dairy. Available isolates from patient stool (n = 1), bulk tank milk (n = 1), and retail milk (n = 1) were identified by CDC as *Campylobacter jejuni* and were indistinguishable by pulsed-field gel electrophoresis (PFGE).

Although the dairy has consistently adhered to PDA requirements for raw milk dairies and conducted milk coliform and somatic cell testing more frequently than required, this was not the first outbreak associated with this dairy. During January–February 2012, the dairy was identified as the source of a multistate outbreak of campylobacteriosis (1). That outbreak was the largest raw milk–associated outbreak in Pennsylvania in the past 2 decades, with 148 associated cases identified. PFGE patterns from the *C. jejuni* strains isolated during the 2012 and 2013 outbreaks differed, consistent with the diversity of *C. jejuni* isolated from cattle on dairy farms (2). PDA also identified *Campylobacter* in bulk tank milk obtained from the dairy during January 2011; no associated human infections were reported.

Repeat outbreaks from raw milk producers are not uncommon and not limited to *Campylobacter*. During 2005–2013, Pennsylvania experienced 17 salmonellosis and campylobacteriosis outbreaks associated with retail raw milk. Five producers had more than one outbreak during that period. Bacterial contamination of raw milk can occur even under optimal conditions; seasonal changes in bovine bacterial shedding or inadequate quality control during milk collection might contribute to outbreak recurrence (2). Findings here and elsewhere indicate that compliance with state regulations and increased producer awareness after an outbreak are insufficient to prevent future outbreaks (3). Public health officials should be vigilant for outbreaks from previously implicated dairies, and public education should stress that avoiding consumption is the most effective way to prevent illness from raw milk products.
